# Molecular physiopathogenetic mechanisms and development of new potential therapeutic strategies in persistent pulmonary hypertension of the newborn

**DOI:** 10.1186/s13052-015-0111-0

**Published:** 2015-02-08

**Authors:** Giuseppe Distefano, Pietro Sciacca

**Affiliations:** Department of Pediatrics, Pediatric Cardiology Service, University of Catania, Via S.Sofia 78, Catania, 95123 Italy

**Keywords:** Persistent neonatal pulmonary hypertension, Pulmonary vascular remodeling, Pulmonary vessels underdevelopment, Lung hypoplasia, Pulmonary vasodilative therapy, Stem cells based therapy

## Abstract

Persistent pulmonary hypertension of the newborn (PPHN) is a cyanogenic plurifactorial disorder characterized by failed postnatal drop of pulmonary vascular resistance and maintenance of right-to-left shunt across ductus arteriosus and foramen ovale typical of intrauterine life. The pathogenesis of PPHN is very complex and can result from functional (vasoconstriction) or structural (arteriolar remodeling, reduced pulmonary vessels density) anomalies of pulmonary circulation. Etiopathogenetic factors heterogeneity can strongly condition therapeutical results and prognosis of PPHN that is particularly severe in organic forms that are usually refractory to selective pulmonary vasodilator therapy with inhaled nitric oxide. This paper reports the more recent acquisitions on molecular physiopathogenetic mechanisms underlying functional and structural forms of PPHN and illustrates the bases for adoption of new potential treatment strategies for organic PPHN. These strategies aim to reverse pulmonary vascular remodeling in PPHN with arteriolar smooth muscle hypertrophy and stimulate pulmonary vascular and alveolar growth in PPHN associated with lung hypoplasia.In order to restore lung growth in this severe form of PPHN, attention is focused on the results of studies of mesenchymal stem cells and their therapeutical paracrine effects on bronchopulmonry dysplasia, a chronic neonatal lung disease characterized by arrested vascular and alveolar growth and development of pulmonary hypertension.

## Introduction

Persistent pulmonary hypertension of the newborn (PPHN), first described as “persistence of fetal circulation” by Gersony and Sinclair in 1969 [1], is a cyanogenic disorder characterized by the lack of postnatal drop of pulmonary vascular resistance and by the persistence of the typical intrauterine right-to-left shunting of blood through foramen ovale and ductus arteriosus. The incidence of PPHN is between 0.43 and 6.6 newborns per 1000 live births and is most common in term and near term newborns [2-4]. Despite the major advances in treatment of newborns with cardiorespiratory diseases, PPHN is still one of the main causes of neonatal death, mortality being around 10-20% [5]. The severe outcome of PPHN is probably linked to the broad spectrum of etiopathogenetic factors some of which can negatively influence therapeutical results. Recent researches on the development of PPHN have shown the important role of perinatal fetal environment (e.g. smoke and drug exposure, stress or pain, maternal obesity and diabetes, caesarean section etc.) plus the epigenetic changes that pre and postnatal stimuli can determine in the expression of genes involved in perinatal pulmonary circulation regulation [6-8].

The purpose of this article is to review the physiopatogenetic aspects of PPHN and underline the molecular mechanisms that can constitute the basis of new potential therapeutical strategies for severe forms of PPHN that are resistant to current treatment.

### Regulation of perinatal pulmonary circulation

During intrauterine life, pulmonary vascular resistance is elevated and systemic resistance low, fetal channels (ductus arteriosus and foramen ovale) are patent with right-to-left shunt and both ventricles work in parallel instead of in series.

Elevated fetal pulmonary vascular resistance is partly caused by pulmonary collapse and vessels tortuosity but above all by pulmonary arterioles vasoconstriction. Normally these arterioles present a muscular medial tunic up to the preacinar zones then disappearing in intraacinar branches [9]. In physiological conditions the periarteriolar muscular layer develops mainly during the last months of gestation and thus is not well represented in preterms [10]. A thicker muscle layer results in a narrower lumen and reduced arterioles compliance, and this may play a role in increased vascular pulmonary resistance, regardless of vasoconstriction [10]. Relative hypoxia in the blood perfusing the fetal lung plays an important role in pulmonary arteriolar vasoconstriction*.* Pulmonary arteriolar muscle fibers are very sensitive to oxygen tension and pH variations and they contract in conditions of hypoxia and acidosis and relax when Pa02 and pH increase [11]. Pulmonary arteriolar tone can also be influenced by several humoral factors present in the perinatal circulation. Some of these (thromboxane, endothelin etc.) possess a vasoconstricting action, whereas others (prostacyclin, nitric oxide, etc.) determine vasodilatation [12,13].

At birth systemic resistance rises rapidly. On the contrary, when breathing starts pulmonary resistance falls after lung and pulmonary vascular bed expansion and, in particular, following arteriolar dilatation caused by the rapid increase of arterial oxygen tension. Oxygen can act directly on myocytes, but its action is mainly mediated by humoral factors (specially prostacyclin and nitric oxide) secreted by the pulmonary arteriolar endothelium, a tissue that performs a key function in perinatal pulmonary circulation regulation [10,12,14,15]. Secretion of these vasodilating agents can also be induced by mechanical stimuli such as ventilation and shear stress caused by vascular bed distension and abrupt increment of pulmonary blood flow [5].

**Figure 1:**
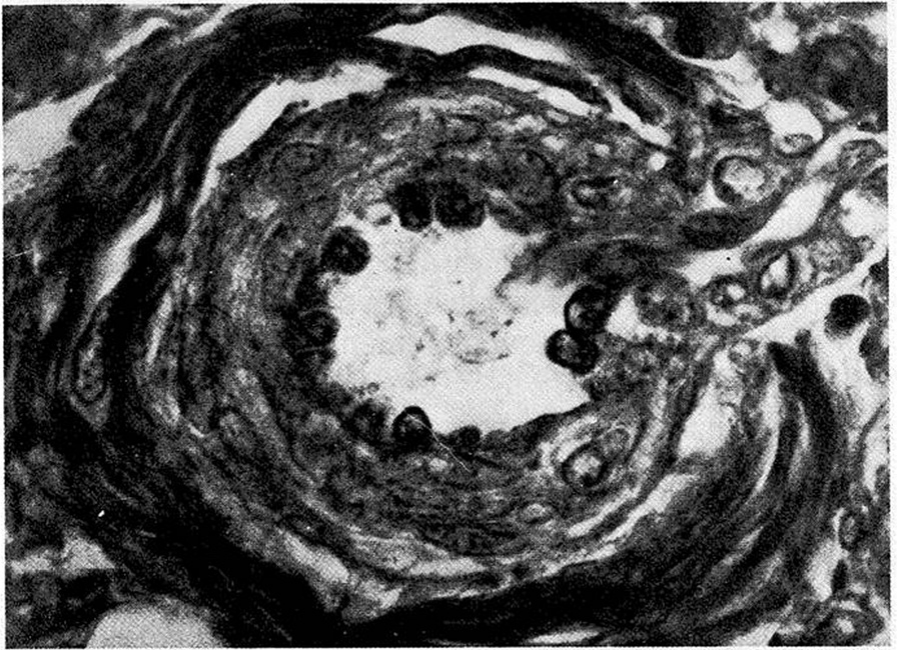
**Pulmonary arteriole from one-day-old infant.** The medial muscle mass is conspicuous (Verhoeff’s and Van Gieson’s stains). From Naeye and Letts [20], Pediatrics 1962.

**Figure 2:**
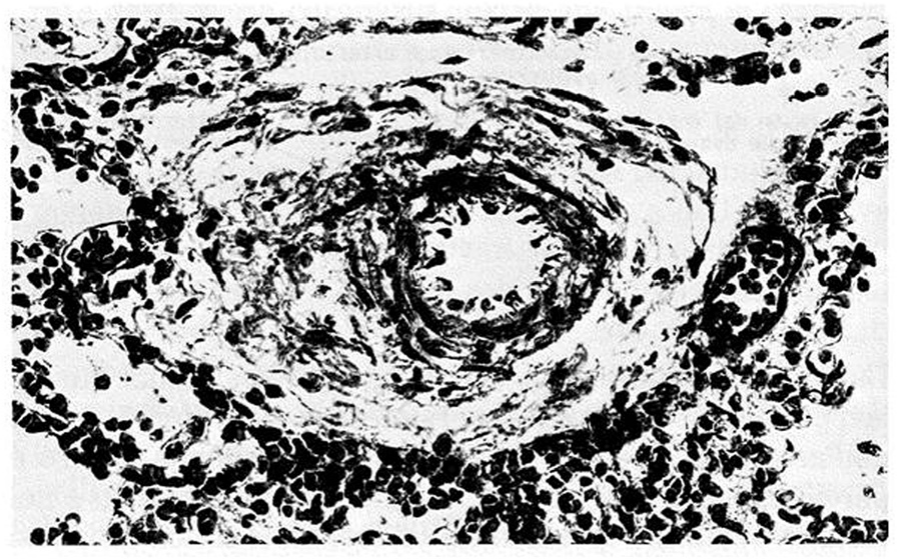
**Pulmonary arteriole from a polymalformed non hypoxemic term infant who died at 6 days of life.** Medial muscle mass is still evident (H.E. stains). From Distefano G et al. [12], Med Surg Ped 1992. (Personal observation).

**Figure 3:**
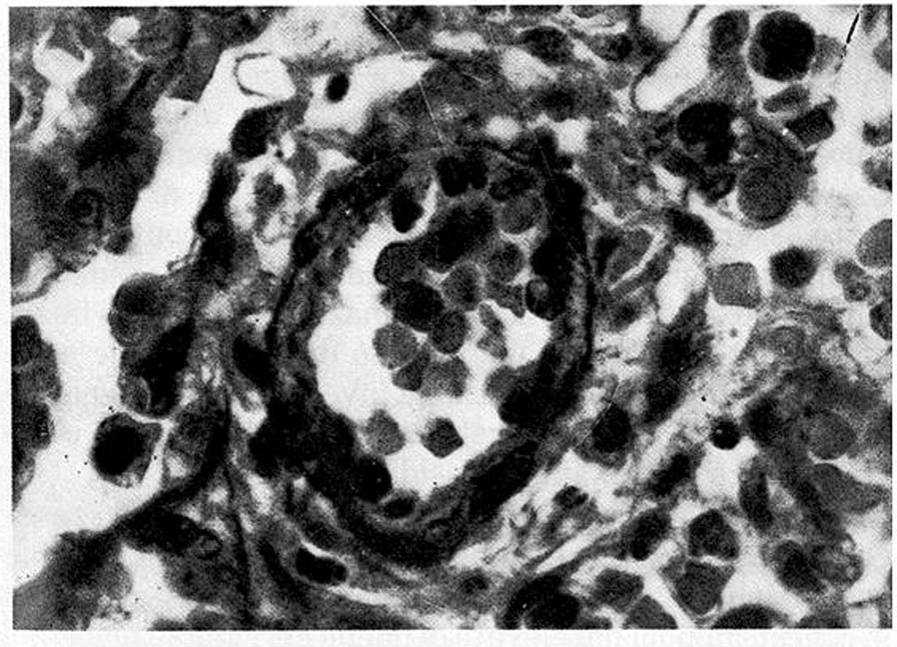
**Pulmonary arteriole from a 4-week-old non hypoxemic infant.** The relative medial muscle mass has decreased since birth (Verhoeff’s and Van Gieson’s stains). From Naeye and Letts [20], Pediatrics 1992.

**Figure 4:**
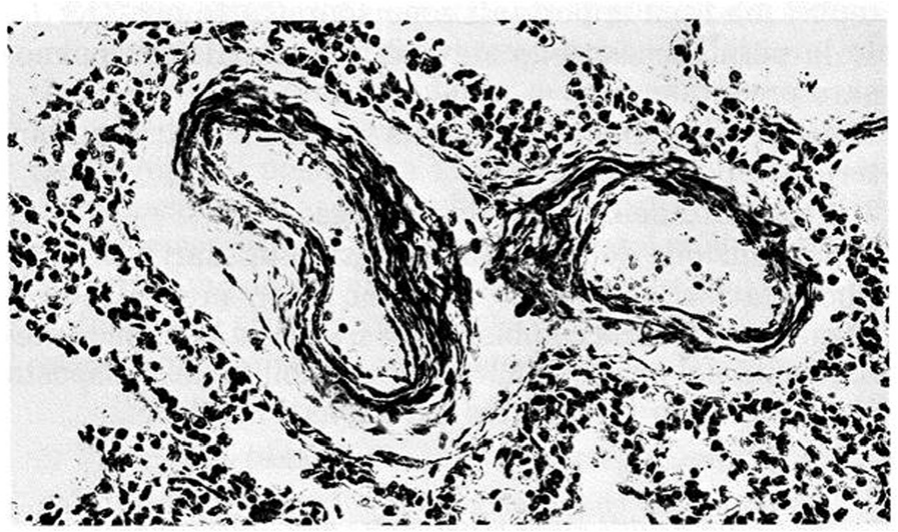
**Pulmonary arterioles from a 22-day-old infant who died from bilateral kidney malformation.** Marked thinning of medial muscle mass is evident (PAS-Gieson stains). From Distefano G et al. [12], Med Surg Ped 1992. (Personal observation).

Nitric oxide (NO) is produced by endothelial NO synthase (eNOS) using L-arginine as substrate and producing L-citrulline as a by-product. L-citrulline in turn can be reconverted in L-arginine through a recycling pathway that is the principal source of L-arginine available to eNOS [16]. eNOS is activated by the sudden increase in postnatal oxygen tension [3]. NO determines pulmonary vasodilatation via soluble guanylate cyclase stimulation and the ensuing increase of cyclic guanylate monophosphate (cGMP) concentrations in vessel smooth muscle cells [17]. cGMP endomyocytic levels are regulated by phosphodiesterase 5 (PDE5), a kinase enzyme abundantly expressed in pulmonary tissue during fetal life and able to hydrolyze cGMP [18]. Pulmonary arterioles vasodilatation at birth is inversely correlated to the thickness that their muscular tunic reaches at the end of gestation**.** Histological observations [19,20,12] revealed that the muscular tunic is progressively reabsorbed during the postnatal period (Figures 1, 2, 3 and 4). This process is sustained by apoptotic events involving pulmonary vessel myocytes and is generally completed within the first two weeks of life. However, sometimes it may take some days longer and thus impact negatively on postnatal circulation adjustments as the muscular tunic thickness narrows the lumen and makes the pulmonary arterioles more reactive to vasoconstricting stimuli [21,22].

### Physiopathology of PPHN

**Figure 5:**
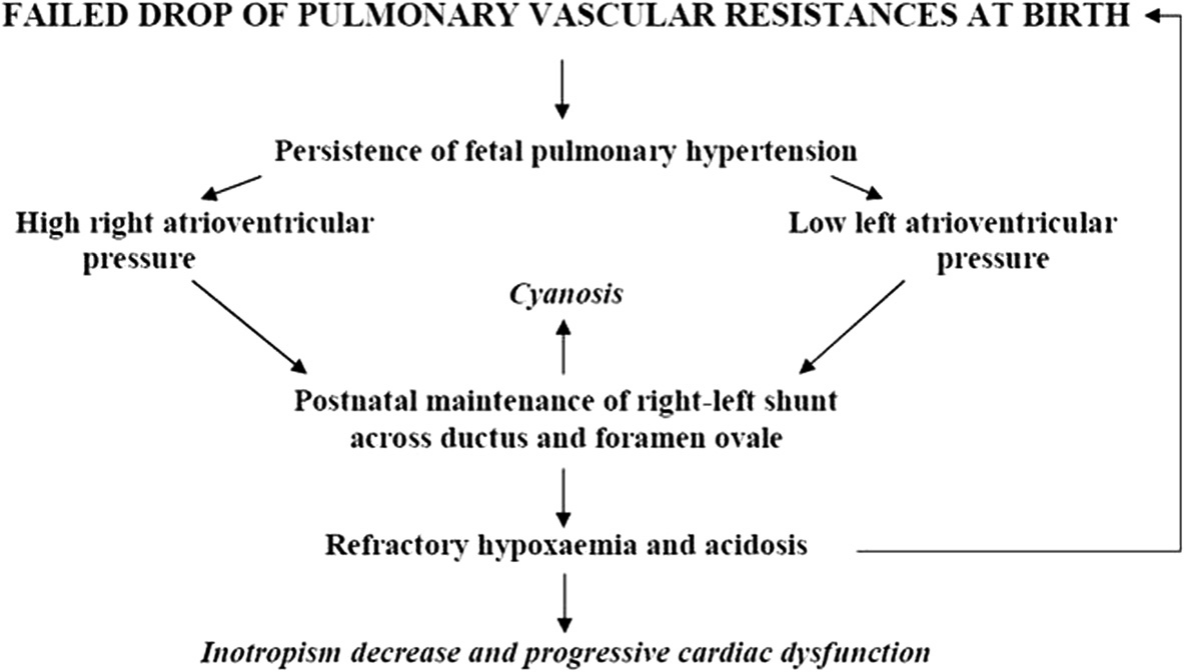
**Physiopathology of PPHN.** Vicious circle leading to progressive cardiac dysfunction.

The fundamental physiopatological feature of this syndrome is the failure of the postnatal drop in pulmonary vascular resistance, and the presence of elevated pressure values in the pulmonary artery and right sections of the heart [7]. This maintains the typical fetal intrauterine right-to-left shunt in the ductus and foramen ovale resulting in cyanosis and severe hypoxemia refractory to oxygen therapy. Hypoxemia and the ensuing metabolic acidosis determine in turn arteriolar vasoconstriction, leading to a further increase in pulmonary vascular resistance, and if this vicious circle is not rapidly eradicated by adequate treatment the negative clinical and hemodynamic situation tends to worsen producing a progressive cardiac dysfunction with fatal outcome (Figure 5).

### Pathogenesis of PPHN

Pathogenetically there are two forms of PPHN: one functional where elevated vascular pulmonary resistance is only due to pulmonary arteriolar vasoconstriction, and the other organic where vasoconstriction is of secondary importance and increased resistance is mainly caused by substantial structural changes in the pulmonary circulation. These changes are generally represented by pulmonary arterioles lumen narrowing caused by muscular tunic hypertrophy and extension of smooth muscle to the intraacinar branches (normally without muscle fibers), or by poor pulmonary vessels development that reduces the size of the pulmonary vascular bed and thus increases blood flow resistance. Organic forms of PPHN include rare, and almost always fatal, cases of alveolar capillary dysplasia caused by diffuse misalignment of the arteriolar capillary venous axis that compromises respiratory exchange [23]. Both functional and organic forms of PPHN can be primary or secondary and have diverse causes [24].

#### Functional PPHN

##### Postnatal persistence of fetal pulmonary vasoconstriction

**Figure 6:**
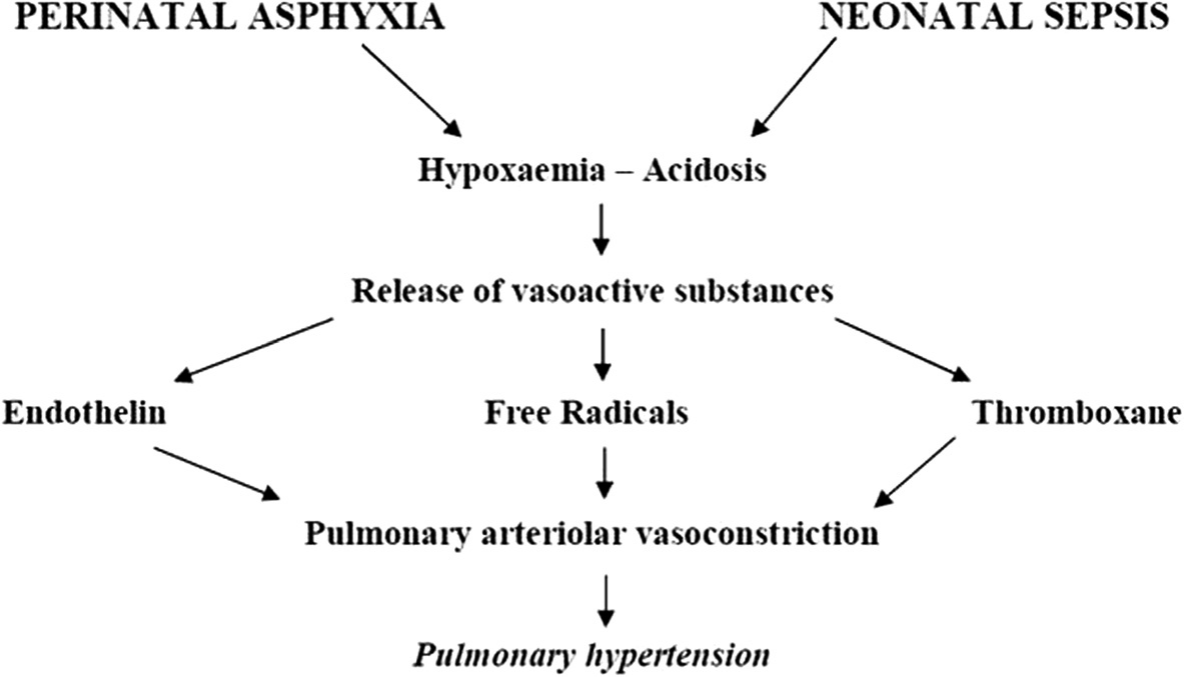
**Pathogenesis of functional PPHN in perinatal asphyxia and neonatal sepsis.**

**Figure 7:**
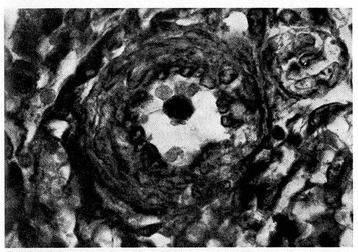
**Pulmonary arteriole from a 6-week-old hypoxemic infant.** The relative medial muscle mass present at birth has been retained (Verhoeff’s and Van Gieson’s stains). From Naeye and Letts [20], Pediatrics 1962.

**Figure 8:**
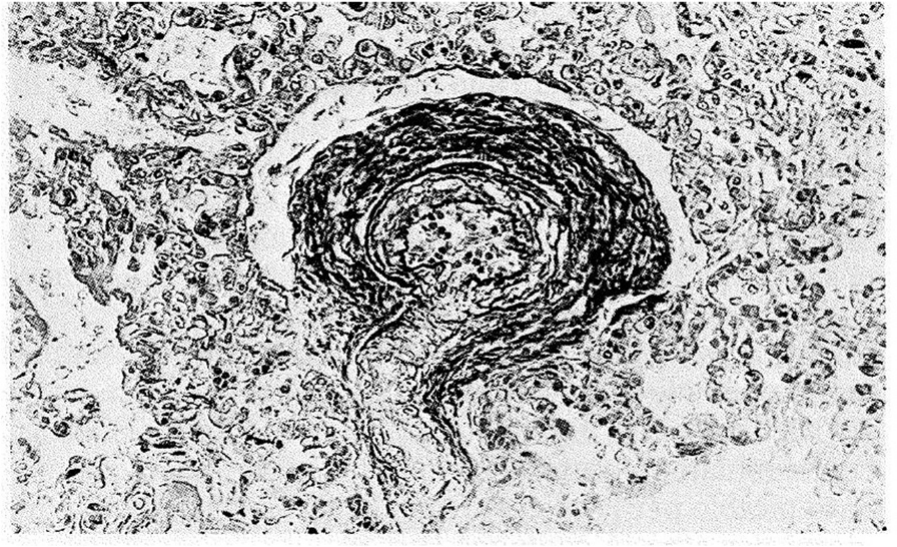
**Pulmonary arteriole from a 11-day-old infant who died from severe perinatal asphyxia.** Marked thickening of medial muscle mass is evident (PAS-Gieson stains). From Distefano G et al. [12] Med Surg Ped 1992. (Personal observation).

Functional PPHN can be idiopathic or, more frequently, secondary to various pathologies. In the idiopathic form, postnatal persistence of fetal pulmonary vasoconstriction is considered the expression of constitutional and/or genetic factors enhancing sensibility of pulmonary arterioles to vasoconstrictive stimuli or impairing mechanisms of NO release and action [25-27]. Reently some PPHN patients have shown variants in the genes of corticotrophin-releasing hormone receptor 1 and of its binding protein (CRHR1 and CRHBP genes). This determines reduced expression of “Peroxisome proliferator-activated receptor-gamma (PPAR-γ)”, a transcription factor that plays an important role in myocytes proliferation and pulmonary arteriolar tone regulation [28-30]. In the secondary form, the majority of cases are due to perinatal asphyxia, present in 80-90% of the subjects, and septic processes caused by group B streptococcus, e.g. pneumonia [1,2]. Asphyxia and sepsis can determine pulmonary arteriolar vasoconstriction, either directly through hypoxia and acidosis or indirectly via release of vasoactive substances, such as leukotrienes, endothelin, free radicals, thromboxane, etc. [31-33] (Figure 6). Thromboxane seems to play a major role in sepsis. In experimentally induced streptococcal infections it was observed that cycloxygenase inhibitors, such as indomethacin, can prevent pulmonary hypertension [34]. Recent studies have revealed that vasoconstriction can also be determined by some phospholipids (in particular, phosphatidyl-glycerol and cardiolipin) present in the bacterial wall of streptococcus B [35]. Studies on perinatal asphyxia have focused on leukotrienes [36] and, especially, on endothelin whose plasma concentrations were markedly higher than observed in normal controls [37]. Reactive oxygen species (ROS), derived from metabolism of hypoxanthine, play a concomitant role in asphyxia [31]. In effect, oxidative stress seems able to enhance signals promoting endothelin production and reducing nitric oxide synthase (NOS) expression [38]. Moreover, recent studies reported that ROS pulmonary vasoconstrictive action can also be mediated by isoprostanes [39]. In perinatal asphyxia, hypoxemia not only contracts pulmonary arterioles but, if prolonged, is also able to hinder muscular tunic reabsorption promoting its hypertrophy as observed in histopathological studies [20,12], (Figures 7 and 8). Similar morphological observations have been reported also in animals with induced postnatal hypoxemia [40]. Among the pulmonary parenchymal diseases associated with perinatal asphyxia - such as meconium aspiration syndrome (MAS), respiratory distress syndrome (RDS) and pneumonia (PM) - the most frequent observed in newborns with PPHN is MAS where endothelin (principally) and urotensin play an important role as powerful pulmonary vasoconstrictive agents [41,42].

#### Organic PPHN

##### Hypertrophy of pulmonary arteriolar muscular tunic

**Figure 9:**
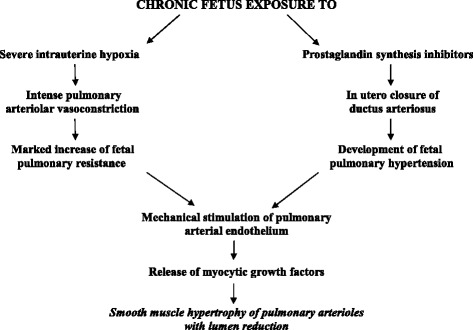
**Pathogenesis of pulmonary vascular remodeling in organic PPHN.**

**Figure 10:**
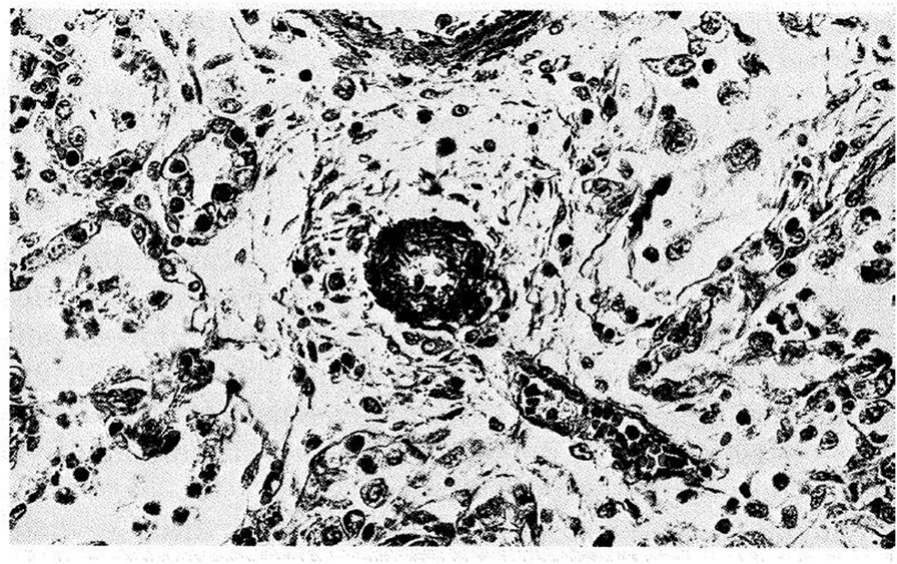
**Pulmonary arteriole from a small for date newborn who died at 2 days of life from esophageal atresia.** Marked thickening of medial muscle mass is evident (H.E. stains). From Distefano G et al. [12], Med Surg Ped 1992. (Personal observation).

The first descriptions of such structural anomaly were reported many years ago in histopathological studies on newborns who died from PPHN and were not affected by neonatal asphyxia or other intercurrent pathologies [43,21,22]. Increased muscular tunic thickness reduces lumen caliber, leading to marked narrowing of the pulmonary arterioles and consequent obstructive type blood flow resistance. As etiology is uncertain, these forms of organic PPHN have been labeled as idiopathic [43]. Some authors believe that genetics may influence these forms of PPHN causing a primitive increased thickness of the muscular tunic of pulmonary arterioles [44]. Other studies have revealed that muscular tunic hypertrophy can be secondary to pathological events occurring during gestation and be observed, for example, in newborns subjected to accentuated chronic intrauterine hypoxia or increase of fetal pulmonary arterial pressure (Figure 9). The first event occurs during intrauterine conditions accompanied by chronic placental insufficiency and can explain the findings of the afore-mentioned vascular changes in small for date newborns [12] (Figure 10). The second event can occur in cases of closure in utero of Botallo’s duct that can occur in newborns of pregnant women treated with prostaglandin synthetase inhibitors (indomethacin, salicylates, lithium etc.) [45] (Figure 9). The important role of chronic hypoxemia and of fetal pulmonary arterial pressure increment in determining smooth muscle hypertrophy in pulmonary arterioles has been demonstrated in experimental models of PPHN reproduced in lamb fetuses [46,47]. Molecular studies revealed that these two factors act on the pulmonary endothelium and can inhibit the release of NO and stimulate that of endothelin and of smooth muscle mitogens inducing pulmonary vessels myocytes hypertrophy [48,49,4,6]. PDE5, an enzyme that degrades cGMP and jeopardizes the pulmonary vasodilating effect of NO, was markedly increased in lamb fetuses with chronic pulmonary hypertension induced by ductus ligation. Recent researchs have shown that the afore-mentioned structural changes in fetal pulmonary arterioles can also occur when there is chronic use of selective serotonin reuptake inhibitors (antidepressant drugs**)** during pregnancy [50,51]. In effect, serotonin is a powerful pulmonary vasoconstrictor that stimulates smooth muscle cell growth and proliferation [50].

##### Underdevelopment of pulmonary vascular bed

In this form of PPHN the failed postnatal drop of pulmonary resistance is due to diminished number of pulmonary vessels that, decreasing the cross-sectional area of pulmonary vascular bed, causes a restrictive type enhancement of resistance to pulmonary blood flow. This severe hemodynamic situation is typical of pulmonary hypoplasia, a condition often associated with congenital diaphragmatic hernia (CDH) [9]. Indeed, it has been clearly documented that normal pulmonary vessel development is crucial for pulmonary alveoli growth and is regulated by various biochemical factors, in particular vascular endothelial growth factor (VEGF), a potent vascular cell mitogen and modulator of angiogenesis [52,53]. Studies on lamb fetuses demonstrated that inhibition of VEGF receptors impairs vascular growth and provokes pulmonary hypertension [54]. Impairment of alveolarization and vascular growth associated with reduced VEGF expression has been reported also in animals with chronic intrauterine pulmonary hypertension induced by ductus ligation [55]. The hypoplastic CDH lung, in addition to reduced vessels density, presents increased vascular reactivity to vasoconstricting stimuli associated with reduced nitric oxide synthase (NOS) expression and elevated endothelin production [23,56,57,6].

### Current medical treatment of PPHN

PPHN treatment is influenced by the multiplicity of etiopathogenic factors, the severity of pulmonary hypertension and by heart and lung function alterations. It includes a general therapy aimed at stabilizing the newborns’ clinical conditions, and a more important specific treatment with pulmonary vasodilators aimed at eliminating the right-to-left shunt causing severe hypoxemia [2,3,7,12,58]. If such treatment fails, extracorporeal membrane oxygenation (ECMO) is required [59]. General therapy,that has been well codified for several years [12,7], and ECMO will not be addressed in this paper.

#### Pulmonary vasodilator therapy

Pulmonary vasodilators are essential in the treatment of PPHN to achieve the reversal of the right-to-left shunt causing hypoxemia and cyanosis.

The advent of nitric oxide (NO) therapy represented a turning-point in the treatment of PPHN. Many clinical and experimental studies have shown that inhaled nitric oxide (iNO), unlike other vasodilators (tolazoline, magnesium sulfate etc.) acting also on the systemic circle [60,3], carries out a selective vasodilating action on pulmonary arterial circulation and eliminates pulmonary hypertension related hypoxemia [61-63]. iNO spreads through the alveolar epithelium to smooth muscle of the underlying vessels and dilates the pulmonary arterioles, while the part that reaches the vessel lumen, through which it could enter the peripheral circulation and cause systemic hypotension, is rapidly inactivated by combination with hemoglobin and formation of methaemoglobin [63]. In PPHN, iNO rapidly improves the oxygenation status and reduces the need for ECMO [64,65,63,2]. In a recent randomized comparative study Gonzalez et al. [66], showed that the best results with iNO were obtained when therapy was administered early on. Nevertheless clinical experience has demonstrated that iNO therapy is effective only in 50–60% of PPHN patients [67,2]. In all likelihood, this depends on the pathogenesis with optimal response to treatment in functional PPHN, that is induced only by pulmonary arteriolar vasoconstriction, and partial or absent response in organic PPHN where structural alterations of pulmonary arterioles sustain stable pulmonary vascular resistance.

PPHN refractory to INO can also be linked to disruptions in the complex down stream signaling pathways activated by the same NO and negatively interfering with its action on vascular myocytes. These changes are sometimes induced by epigenetic alterations and include various anomalies, such as reduced response of guanylate cyclase to NO, reduced expression or activity of guanylate cyclase, increased clearance of cGMP by phosphodiesterase [68]. In cases of resistant PPHN to INO therapy, an alternative treatment could be the use of PDE inhibitors that preventing cGMP degradation and raising its intramyocytic concentration, promote pulmonary arterioles vasodilation [63,2]. One of these substances, the powerful PDE5 inhibitor sildenafil, has attracted most attention**.** PDE5 is the most expressed isoform in the lung during the perinatal period and its activity is increased in PPHN animal models [7]. Oral or intravenous administration of sildenafil is able to lower pulmonary vascular resistance with poor impact on systemic resistance in experimental and clinical studies [69-71]. Some studies revealed its efficacy also in cases of PPHN associated with congenital diaphragmatic hernia where it can improve pulmonary vascular function and promote pulmonary growth [72]. Furthermore it has been reported that this drug enhances vasodilative response to endogenous nitric oxide and thus prevents pulmonary hypertension rebound after the suspension of nitric oxide therapy [70]. Adenosine, an eNOS agonist, could increase the formation of N0 and because of its extremely short half-life, it may have fewer systemic side effects. However, the experience using this substance in PPHN is still very limited [3]. Additional pulmonary vasodilatory effect in cases of PPHN not responding well to INO therapy has been reported using inhaled prostacyclin **(**PGI2) and intravenous infusion of milrinone. These substances trigger off complementary effects on pulmonary vascular myocytes to those obtained by NO as they raise cAMP intracellular concentrations. PGI2 directly stimulates adenil cyclase, while milrinone acts indirectly by inhibiting PDE3, a cAMP hydrolyzing enzyme. However, to date controlled studies confirming their efficacy are lacking [63,3]. A recent randomized, double-blind, placebo-controlled, prospective study in 47 newborns infants with PPHN showed that Bosentan, an endothelin-1 antagonist, is an efficacious pulmonary vasodilator [73].

Despite progress in PPHN therapy, this syndrome has still a severe prognosis. Clearly, the major problems involve newborns presenting structural changes in pulmonary circulation, such as muscle hypertrophy and pulmonary vascular bed hypoplasia that reduce the efficacy of vasodilator therapy also when it acts selectively on pulmonary vessels. Advances in molecular pathogenetic studies seem to offer new potential therapeutic solutions for these severe cases of PPHN.

### New potential therapeutical strategies

Increasing knowledge of complex molecular mechanisms involved in the muscularization process of pulmonary arterioles and the development of pulmonary vascular bed, forms the basis for new therapeutic strategies aimed at promoting periarteriolar muscle involution in PPHN associated with pulmonary vascular remodeling, and at stimulating angiogenic processes in PPHN associated with underdevelopment of pulmonary vasculature.

**Figure 11:**
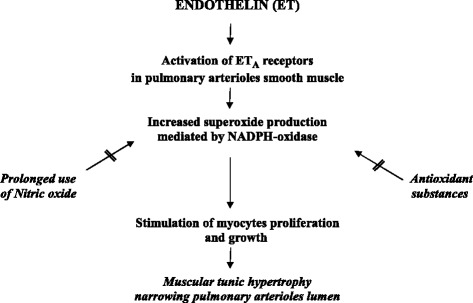
**Role of Endothelin in the pathogenesis of pulmonary vascular remodeling in organic PPHN.** In animal models of PPHN the negative effect of superoxide can be counteracted by Nitric oxide-donors and L-citrulline enhancing, respectively, exogenous and endogenous Nitric oxide bioavailability (see text).

A major role in pulmonary vascular remodeling seems to be played by endothelin-1 (ET-1) whose concentrations are increased in newborns with PPHN. ET-1 not only has a well known vasoconstrictive action, but also stimulates proliferation of pulmonary arterial smooth muscle cells (PASMCs), as observed by Wedgwood *et al*. [74] in *in vitro* studies on PASMCs isolated from lamb fetuses. These effects are linked to ETa receptors activation in the PASMCs; in fact blockade of these receptors attenuated fetal pulmonary hypertension and inhibited pulmonary periarteriolar musculature hypertrophy in ovine models where PPHN was induced by ductal ligation [74]. Furthermore, these authors observed that the mitogenic effect on PASMCs linked to ETa receptors stimulation was mediated by NADPH oxidase through increased production of superoxide, that in turn stimulate activation of mitogen-activated protein(MAP) kinases, and can be prevented by antioxidant treatment and by NADPH oxidase inhibition [75] (Figure 11). In further studies on lamb fetuses the same authors [76] reported that PASMACs proliferation induced by ET-1 via the superoxide can be inhibited by both endogenous and exogenous NO. Using the NO donor spermine NONOate they observed that nitric oxide released by this substance, reacting with superoxide to form peroxynitrite, lowers superoxide levels preventing its stimulating effect on PASMCs proliferation and viability and can also determine apoptosis of the same PASMCs through the activation of pro-apoptotic caspases enzymes [76]. Increased superoxide production in pulmonary arteries and elevated ET-1 concentrations associated with reduced NO production have been found in lambs with chronic ductal ligation-induced PPHN [77,78]. Such experimental data indicate that prolonged administration of NO, also through use of NO donors, combined with antioxidant therapy could be a useful therapeutic tool to reverse smooth muscle hypertrophy in severe forms of PPHN associated with pulmonary vascular remodeling. In order to increase NO availability, some authors have taken into consideration therapeutical use of L-citrulline. This substance in fact, is capable of enhancing endogenous nitric oxide production via its action that improves pulmonary eNOS function [16]. Recently a study in newborn piglets exposed to 10 days of chronic hypoxia showed that pulmonary hypertension development can be prevented by supplementation of L-citrulline [79]. In children undergoing cardiopulmonary bypass for cardiosurgery it has been observed that L-citrulline supplementation was able to prevent postoperative pulmonary hypertension [80]. If these results are confirmed by a larger series of studies, the use of L-citrullin could be a useful remedy for preventing the development of pulmonary hypertension and vascular remodeling in neonates affected by pathologies associated with prolonged hypoxia.

Regarding PPHN with reduced pulmonary vascular bed it is known that the syndrome is typical of the pathological conditions associated with pulmonary hypoplasia, in particular congenital diaphragmatic hernia (CDH) [9]. Reduced pulmonary vessel density has also been observed in clinical and experimental forms of PPHN secondary to chronic intrauterine fetal pulmonary hypertension [55,7].

Following embryonic vasculogenesis where primordial vascular structures are formed, pulmonary circulatory network development is induced by angiogenesis a biological process regulated by a series of transcription and growth factors, in particular VEGF that is paramount in pulmonary vessel growth [13]. In the human fetal lung, VEGF is expressed in epithelial cells and myocytes while its receptors are located in endothelial cells closely apposed to the developing epithelium [81]. VEGF’s angiogenic effect on pulmonary endothelium is mediated by NO produced by the activation of endothelial nitric oxide synthase (eNOS) [82]. Reduced VEGF expression with impaired nitric oxide signals has been reported in experimental PPHN models associated with chronic fetal pulmonary hypertension after in utero ductal ligation, and with nitrofen-induced diaphragmatic hernia. Both these conditions present vascular remodeling and reduced pulmonary vessel density [54,83]. NO administered to rats with nitrofen-induced CDH to stimulate angiogenesis enhanced lung growth [13]. The results of this research suggest that cases of PPHN associated with reduced pulmonary vessels development could benefit from prolonged treatment with iNO or with NO donors. Benefits would be two-fold as such therapy could determine the regression of periarteriolar muscular hypertrophy, and also stimulate pulmonary angiogenesis. On the other hand, reduced eNOS expression with low blood levels of NO has been reported in human newborns with diaphragmatic hernia [56] and in experimental animal models with PPHN associated with chronic hypoxia and pulmonary hypertension [84,85]. Recently Teng et al. performed in vitro studies on pulmonary artery endothelial cells (PAEC) isolated from lambs with intrauterine ductal ligation-induced PPHN and showed that angiogenesis can be strongly stimulated by sepiapterin, a substance capable of increasing intracellular levels of tetrahydrobiopterin (BH4**)** that are low in these cells. BH4 is a cofactor of critical importance to maintain active eNOS catalytic function for NO production, and in PAEC of these PPHN lambs can be inactivated by its oxidation in dihydrobiopterin (BH2), due to increased peroxynitrite formation in these cells [86]. Moreover, in their previous study using the same PPHN lamb model, Teng et al. [87] showed that impaired angiogenesis in PAEC improved after addition of antioxidants on tissue culture media. The results of such researches suggest that the use of sepiapterin to increase PAEC BH4 intracellular levels might be a potentially useful therapy for improving eNOS function and restoring angiogenesis in PPHN cases with reduced density of pulmonary vessels. Furthermore, the effectiveness of this treatment could be potentiated by combining sepiapterin supplementation and antioxidant therapy.

### Future perspectives in PPHN with lung hypoplasia

In this field, very interesting therapeutical perspectives are emerging from experimental studies on bronchopulmonary dysplasia (BPD), a chronic neonatal lung disease characterized by arrested vascular and alveolar growth and development of pulmonary hypertension. They indicate the potential use of mesenchymal stem cells (MSCs) or endothelial progenitors cells (EPCs) for restoring vascular and alveolar growth [88,89]. MSCs are multipotent cells capable of self-renewal and differentiating into various cell types, including parenchymal and vascular pulmonary cells, and can be obtained from bone marrow (BM), umbilical cord blood (UCB) and adipose tissue [89]. EPCs are BM or UCB-derived vascular precursor cells which can be circulating and also resident within vessels wall. Recently several studies In various animal models of BPD have demonstrated that intravenous or intratracheal delivery of bone marrow-derived MSCs (BMSCs) was capable of regenerating lung vascular and alveolar growth and reversing associated pulmonary hypertension [90]. The same effects in BPD models have been obtained with human UCB-derived EPCs [91]. However, a very important acquisition emerging from stem cells studies is that, rather than cells replacement, the beneficial therapeutic effect of BM and UCB-derived MSCs or EPCs can be mediated through a paracrine mechanism [92]. This possibility is suggested by low intrapulmonary engraftment rates of these transplanted cells and supported by in vitro and in vivo studies showing that cell-free conditioned media, derived from cultures of these cells, prevent and/or restore arrested alveolar and vascular growth in neonatal rodents models of lung injury induced by chronic hyperoxia [92]. MSCs-derived conditioned media are rich in soluble factors such as VEGF, stanniocalcin-1 (a potent antioxidant) and specially exosomes**,** microvesicles containing microRNAs molecules and other bioactive molecules involved, respectively, in gene expression regulation, intercellular communication signals and the control of inflammatory response. These factors can protect the lung from injuries inducing alveolar and vascular damage and stimulate proliferation and differentiation of resident epithelial and endothelial progenitors cells and restore pulmonary growth [92,93]. Recently, the therapeutical importance of exosomes has been demonstrated by Lee et al. [94] in the rodent model of chronic hypoxia-induced pulmonary hypertension. In this model the intravenous delivery of both animal or human MSC-derived exosomes inhibited vascular remodeling and development of pulmonary hypertension; on the contrary, no therapeutical effect was obtained by removing exosomes from MSCs-conditioned media.

## Conclusions

PPHN is a plurifactorial syndrome with a complex pathogenesis sustained by functional (vasoconstriction) or structural (periarteriolar muscular hypertrophy, reduced pulmonary vessels density) pulmonary circulation anomalies. Despite considerable therapeutical progress achieved using inhaled nitric oxide (INO), a selective pulmonary vasodilator, PPHN still remains a major cause of mortality in all neonatal centers. Prognosis is particularly severe in organic forms, that are refractory to INO and other alternative pulmonary vasodilators such as sildenafil and prostacyclin. Recent advances, in the understanding of molecular physiopathogenetic mechanisms, have paved the way for new potential therapeutical strategies for these organic forms that, as observed in animal models, could benefit from prolonged use of nitric oxide donors (NO-donors) and of L-citrulline and sepiapterin. These substances are capable of increasing, respectively, the exogenous and endogenous availability of NO. In PPHN with pulmonary vascular remodeling, increased circulatory levels of NO can reverse periarteriolar muscular hypertrophy counteracting the stimulating effect of endothelin-1 (whose concentrations are elevated in these cases) on pulmonary myocites proliferation and growth. While in PPHN with reduced density of pulmonary vessels, high NO levels can stimulate vascular growth mediating angiogenic effect of VEGF on pulmonary endothelial cells.

Nevertheless, regarding more severe PPHN associated with lung hypoplasia (as in congenital diaphragmatic hernia), very fascinating future therapeutical perspectives are emerging from experimental studies on bronchopulmonary dysplasia (BPD). Such indicate the possible use of human umbilical cord blood-derived mesenchymal stem cells (UCB-derived MSCs) and/or of bioactive factors obtained by their cultures to restore vascular and alveolar growth and reverse pulmonary hypertension. Recently, it has been shown that some of these factors (exosomes) are, also, effective in inhibiting vascular remodeling and pulmonary hypertension development in rodents models of hypoxia-induced PPHN. If the beneficial results achieved in experimental models are confirmed by further controlled studies and reproduced in human newborns, it is reasonable to postulate that, in near future, the use of bioactive substances obtained by cultures of human UCB-derived MSCs (easily accessible at birth) could revolutionize the prognosis of severe organic PPHN.
